# Submillisecond-Response Polymer Network Liquid Crystal Phase Modulators

**DOI:** 10.3390/polym12122862

**Published:** 2020-11-30

**Authors:** Yannanqi Li, Zhiyong Yang, Ran Chen, Lingchao Mo, Juanli Li, Minggang Hu, Shin-Tson Wu

**Affiliations:** 1 College of Optics and Photonics, University of Central Florida, Orlando, FL 32816, USA; yannanqili@Knights.ucf.edu (Y.L.); zhiyyang@Knights.ucf.edu (Z.Y.); 2 Key Laboratory of Applied Surface and Colloid Chemistry, School of Materials Science and Engineering, Shaanxi Normal University, Xi’an 710065, China; tradchenr@snnu.edu.cn; 3 Optical and Electrical Material Center, Xi’an Modern Chemistry Research Institute, Xi’an 710065, China; xiaomo3056@126.com (L.M.); lijl208@sohu.com (J.L.)

**Keywords:** submillisecond-response, polymer network liquid crystal, phase modulators

## Abstract

A submillisecond-response and light scattering-free polymer-network liquid crystal (PNLC) for infrared spatial light modulators is demonstrated. Our new liquid crystal host exhibits a higher birefringence, comparable dielectric anisotropy, and slightly lower visco-elastic constant than a commonly employed commercial material, HTG-135200. Moreover, the electro-optical performance of our PNLCs with different monomer concentrations, cell gaps, and liquid crystal (LC) hosts is compared and discussed from four aspects: operating voltage, hysteresis, relaxation time, and light scattering loss. The temperature effect on hysteresis is also analyzed. Potential applications of PNLCs for laser beam steering and spatial light modulators especially in the infrared region are foreseeable.

## 1. Introduction

Spatial light modulators, such as liquid-crystal-on-silicon (LCoS) [1,2,3], have found widespread applications in adaptive optics [4,5,6,7], holographic near-eye display [8,9], laser beam steering [10,11,12,13], time-multiplexing 3D displays [14], and adaptive lens [15,16]. Unlike an amplitude modulator, a reflective LCoS usually requires 2*π* phase change, δ = 2(2*πd*Δ*n*/*λ*), where the first factor 2 represents the double-pass due to reflective mode, *d* is the cell gap, Δn is the LC birefringence, and *λ* is the wavelength. In the visible region, to achieve 2*π* phase change demands *d*Δ*n* = *λ*/2, which can be achieved using a high Δ*n* and low viscosity nematic LC [17,18]. As the wavelength increases to infrared (IR), to maintain 2π phase change, a thicker cell gap for compensating the decreased Δ*n* and longer wavelength (λ) is needed. This will dramatically increase the response time because the response time of a LC device is proportional to *d*^2^. To reduce response time, a multi-layer approach [19,20] has been elegantly demonstrated but the tradeoff is its complicated and slow fabrication process. Polymer network liquid crystal (PNLC) can also be viewed as a multi-layer structure partitioned by polymer networks [21] for achieving fast response time while keeping 2π phase change, especially in the mid-wavelength infrared region [22,23,24,25,26]. 

Typically, a PNLC precursor consists of ~93% nematic LC host, ~6% reactive mesogen monomer, such as RM257, and ~1% photo-initiator. Upon UV curing, submicron polymer network domain size is formed to constrain LC molecules. Thus, submillisecond response time can be achieved due to small LC domain size and strong anchoring energy provided by the networks. However, the tradeoffs include high operation voltage, hysteresis, double relaxation, and light scattering loss. In a PNLC, the operating voltage is determined by several factors, such as dielectric anisotropy (Δε) and Δ*n* of the LC host, cell gap, and domain size. A LC material with large Δ*ε* and high Δ*n* is desirable because it enables a thinner cell gap to be used, which in turn helps lower the required voltage. Reducing the monomer concentration will lead to a lower $V_{2\pi }$ (the voltage with 2π phase change), but the increased domain size would cause a slower response time, double relaxation, and light scattering. HTG-135200 is a commercial LC developed for polymer-stabilized LC devices, including blue phases [27], because of its relatively high Δn (≈0.2) and large Δε (≈70−80). As pointed out in [28], the measured Δε value of HTG-135200 could vary by 33%, depending on whether the voltage shielding effect of alignment layers is taken into consideration.

In this paper, we report a new high Δn nematic LC (called M1, synthesized and formulated by Xi’an Modern Chemistry Research Institute) for making PNLCs and characterize their electro-optical performance, including $V_{2\pi }$, hysteresis, response time, and light scattering loss. Good agreement between experimental results and theoretical analysis is obtained. Further, the temperature effects on hysteresis are discussed. Compared to HTG-135200, our LC exhibits a higher Δn and slightly larger Δε without compromising viscosity. Under the same monomer concentration and cell gap, our PNLC offers a lower $V_{2\pi }$ than that using HTG-135200. Hysteresis, relaxation time, and light scattering loss are also compared and discussed for PNLCs using these two hosts.

## 2. Materials Characterization

The physical properties of M1 and HTG-135200 (abbreviated as HTG) are measured at 25 °C and results are listed in Table 1. The clearing point ($T_{c}$) was measured by a Differential Scanning Calorimetry (DSC, TA instruments Q100) and Δε was measured by a multi-frequency LCR meter HP-4274. Compared to HTG, M1 exhibits ~10% higher Δn at *λ* = 1.06 μm and slightly larger Δε at 1 kHz. Large Δ*ε* helps to lower the threshold voltage ($V_{th}$) and $V_{2\pi }$. Once Δ*ε* is obtained, the splay elastic constant $K_{11}$ can be calculated from $V_{th}$ [29,30]:

(1) $$V_{th}=\pi \sqrt{\frac{K_{11}}{\varepsilon_{0}\Delta \varepsilon }}$$

In Equation (1), $\varepsilon_{0}$ is the vacuum permittivity, and $V_{th}$ can be measured from the voltage-dependent transmittance (VT) curve. Consequently, the rotational viscosity ($\gamma_{1}$) can be extracted from the measured visco-elastic constant (*γ*_1_/*K*_11_) from the free relaxation time. 

### 2.1. Birefringence

**Figure 1:**
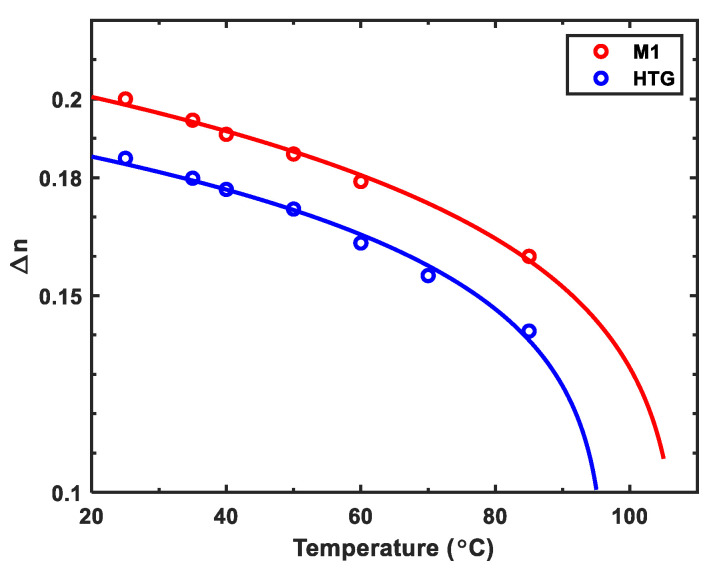
Temperature-dependent birefringence of M1 and HTG at *λ* = 1.06 μm and 1 kHz. Dots are experimental data and solid lines are fitting curves with Equation (2).

To measure Δn at different temperatures and wavelengths, we first injected M1 and HTG into commercial homogenous cells with cell gap *d* = 8 μm. The pretilt angle of the rubbed polyimide alignment layers is about 3 °C. Then the cell was fixed on a Linkam heat stage controlled by TMS94 Temperature Programmer and sandwiched between two crossed polarizers. The Δ*n* at each temperature is obtained from the measured phase retardation by applying a 1 kHz square-wave AC voltage to the LC cell. Figure 1 depicts the temperature-dependent Δ*n* at *λ* = 1.06 μm, where dots represent the measured data and solid line is the fitting curve with Haller’s semi-empirical equation [31]:

(2) $$\Delta n=\Delta n_{0}S=\Delta n_{0}{\left(1-T/T_{c}\right)}^{\beta }.$$

In Equation (2), $\Delta n_{0}$ represents the extrapolated birefringence at *T* = 0 K, *S* is the order parameter, and *β* is a material constant. The obtained $\Delta n_{0}$ and β values are listed in Table 2. From Figure 1, we can see that the Δn of M1 is about 10% higher than that of HTG in the 20 to 100 °C range. 

### 2.2. Visco-Elastic Constant

**Figure 2:**
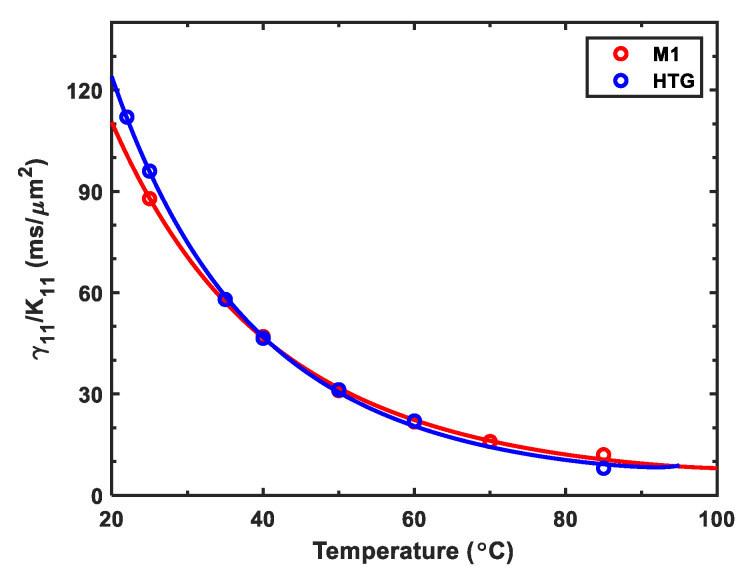
Temperature-dependent visco-elastic constant of M1 and HTG. Dots are measured data and solid lines are fitting curves with Equation (3). The fitting parameters are listed in Table 2.

The visco-elastic coefficient ($\gamma_{1}/K_{11}$) of an LC material determines the response time and the rate of polymer network formation [22]. By measuring the transient decay time of M1 and HTG LC cells, we obtained their $\gamma_{1}/K_{11}$. Figure 2 depicts the $\gamma_{1}/K_{11}$ at different temperatures, in which dots represent the measured data and solid lines represent fitting curves with following equation [32]:

(3) $$\frac{\gamma_{1}}{K_{11}}=A\frac{\exp\left(E_{a}/k_{B}T\right)}{{\left(1-T/T_{c}\right)}^{\beta }}.$$

In Equation (3), *A* is a proportionality constant, $E_{a}$ is the activation energy, and $k_{B}$ is the Boltzmann constant. The fitting parameters *A* and $E_{a}$ are included in Table 2. From Figure 2, we find that these two LCs possess a comparable $\gamma_{1}/K_{11}$. As the temperature increases, $\gamma_{1}/K_{11}$ decreases dramatically. 

### 2.3. Wavelength Dispersion

**Figure 3:**
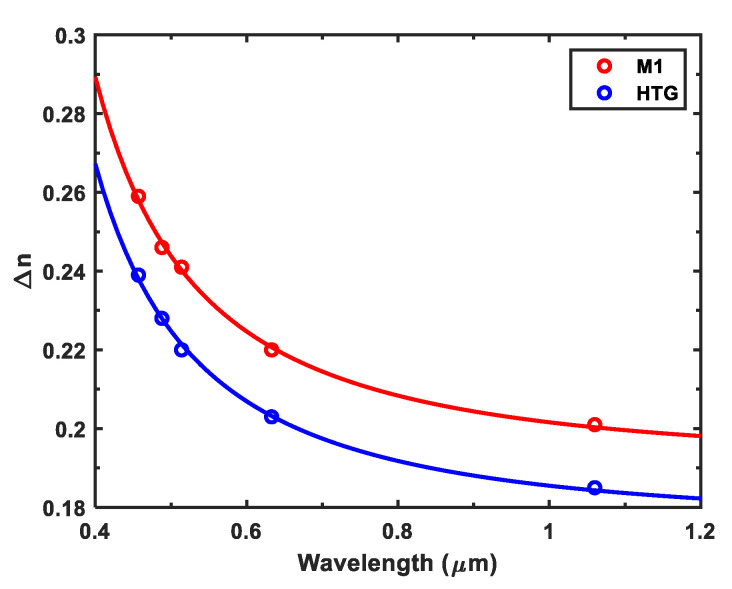
Wavelength-dependent Δn of M1 and HTG. Dots are measured data and solid lines are fitting curves with Equation (4). The fitting parameters are listed in Table 2.

To investigate the device performance at different wavelength, the birefringence dispersion of these two LC hosts should be measured. The probing beams we employed include a diode laser at *λ* = 1.06 μm, a He-Ne laser at *λ* = 632.8 nm, and a tunable Argon ion laser (*λ* = 457, 488, and 514 nm). The experimental results are shown in Figure 3, where dots represent the measured data and solid lines are the fitting curves with the single-band birefringence dispersion equation [33]:

(4) $$\Delta n=G\frac{\lambda^{2}\lambda^{*2}}{\lambda^{2}-\lambda^{*2}}.$$

In Equation (4), G is a proportionality constant and λ* is the mean resonance wavelength. Once these two parameters are determined, the birefringence at any wavelength of interest can be calculated from Equation (4). The obtained G and λ* values are also listed in Table 2. According to Equation (4), Δn is reduced to $G\lambda^{*}{}^{2}$ when $\lambda \gg \lambda^{*}$; that is to say, in the long wavelength region Δn reaches a plateau. The extrapolated $G\lambda^{*}{}^{2}$ value of M1 and HTG is 0.191 and 0.176, respectively.

## 3. Polymer Network Liquid Crystals

To fabricate transmissive-mode PNLCs, we first prepared precursors by adding different amounts of reactive mesogen RM257 (Merck) and 0.5 wt% photo-initiator Irgacure 819 to M1 and HTG LC hosts. Next, we filled each precursor into ~10 μm and 12.15 μm homogeneous LC cells (glass substrates) whose inner surface was deposited with a thin indium-tin-oxide (ITO) electrode and then overcoated with a polyimide alignment film. Then, a UV lamp (λ ≈ 365 nm at intensity ≈ 35 $\mathrm{mW}/{\mathrm{cm}}^{2}$) was used to cure the samples at 25 °C for 40 min. As listed in Table 3, we have prepared 8 samples for comparison. In PNLCs, to get a firm polymer network and fast response time, the monomer concentration is usually around 6%. To explore the monomer effect on the electro-optical performance of PNLCs, we choose two different monomer concentrations: 5.7% and 6.6%, as listed in Table 3. Regarding to cell gap, we need to consider whether it provides the required 2*π* phase change at a reasonable voltage. However, the cell gap merely satisfies 2*π* phase change will lead to a very high *V*_2*π*_. To lower *V*_2*π*_, we choose a slightly thicker cell gap to achieve about 2.2*π* phase change, which corresponding to *d* ≈ 10 to 12 μm, depending on which LC host is employed. If the cell gap is too thick, then the light scattering loss will increase proportionally.

In the following sections, the LC host, monomer concentration, and cell gap effects on VT curves, hysteresis, relaxation time, dielectric relaxation, and light scattering loss will be discussed in detail. 

### 3.1. Voltage-Dependent Phase Change 

**Figure 4:**
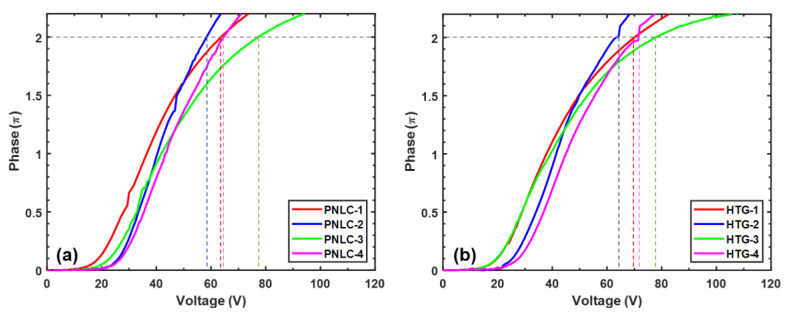
Voltage-dependent phase change curves of 8 transmissive PNLC samples at *λ* = 1.06 μm: The LC host of (**a**) PNLC 1–4 is M1, and (**b**) HTG 1–4 is HTG. The operating temperature is 25 °C. The vertical dashed lines indicate the $V_{2\pi }$ values for different samples.

Figure 4 shows the voltage-dependent phase change (VP) curves for the eight transmissive PNLC samples we prepared. Unlike a nematic LC device, according to Sun’s multi-layer model [23], the threshold voltage of a PNLC is proportional to the cell gap as

(5) $$V_{on}\propto \frac{\pi d}{d_{1}}\sqrt{\frac{K_{11}}{\varepsilon_{0}\Delta \varepsilon }\;}.$$

In Equation (5), $d_{1}$ is the average domain size. Therefore, for a given LC host, $V_{on}$ is mainly determined by the cell gap and domain size. The domain size can be controlled by the monomer concentration and diffusion rate. A thicker cell gap or a smaller domain size would lead to a higher $V_{on}$. This is confirmed by the VP curves of PNLC-4 and HTG-4 in Figure 4. 

For a given wavelength *λ*, to achieve 2*π* phase change using a transmissive PNLC, the cell gap (*d*) is determined by the effective birefringence Δ*n_eff_* of the PNLC composite as

(6) $$d=\lambda /\Delta n_{eff}$$

From Equation (6), a high Δ*n* LC host helps to increase the Δ*n_eff_* of PNLC, which in turn enables a thinner cell gap to be used. By substituting Equation (6) into Equation (5), $V_{2\pi }$ can be expressed as

(7) $$V_{2\pi }\sim \frac{\lambda }{\Delta n_{eff}d_{1}}\sqrt{\frac{K_{11}}{\varepsilon_{0}\Delta \varepsilon }}$$

Equation (7) shows that high Δ*n_eff_* and large Δ*ε* help to lower $V_{2\pi }$. In our experiment, the working wavelength is *λ* = 1.06 μm. The measured $V_{2\pi }$ values of the 8 PNLC samples are listed in Table 3. Because our M1 host has a higher Δn and larger Δ*ε* than HTG, under the same cell gap and monomer concentration, the $V_{2\pi }$ of PNLC-4 is lower than that of HTG-4. Meanwhile, as Table 3 shows, under the same monomer concentration and cell gap, M1-based PNLCs offer a lower *V*_2*π*_ than the corresponding HTG host, as clearly shown by comparing PNLC-1 with HTG-1, PNLC-2 with HTG-2, and PNLC-3 with HTG-3. The reason is that M1 has a 10% higher birefringence, while not compromising its dielectric anisotropy and viscosity. Our employed LC cells have a small variation in cell gap, but the overall trend of VP curves is consistent with our analysis. In Figure 4, some of the measured VP curves are not very smooth, which is attributed to the intensity fluctuation of our light source. However, such a small fluctuation will not affect the measured results. Overall, we find that a larger dΔn and larger domain size help to reduce $V_{2\pi }$. However, a thicker cell gap would lead to a more noticeable light scattering loss, and a larger domain size would cause a slower relaxation time. The trade-off between balancing light scattering loss and relaxation time by choosing different cell gap and monomer concentration will be discussed in the following sections. 

### 3.2. Relaxation Time

**Figure 5:**
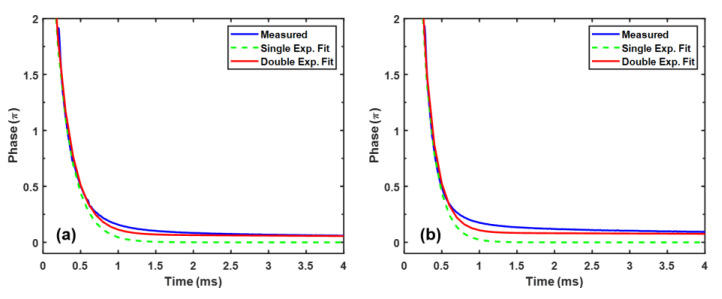
Time-dependent phase change curves of PNLC-4 and HTG-4 at λ=1.06 μm and 25 °C: (**a**) PNLC-4, and (**b**) HTG-4.

In a polymer-stabilized LC system, when the applied voltage is removed, the process of relaxing back to the original state sometimes cannot be simply described by a single exponential decay because of the sophisticated interaction between LC and polymer network. Usually, the relaxation process involves two steps, starting with a fast decay from the submicron LC domains and then followed by a slow relaxation caused by the electrostriction effect of polymer network [34]. Such a two-step relaxation process is called double relaxation. To investigate the relaxation time, we choose samples PNLC-4 and HTG-4 to conduct the experiments because both samples have 6.6% monomer concentration. The transient phase change is recorded, as Figure 5 shows, by instantaneously removing the biased $V_{2\pi }$. The measured relaxation time is calculated between 90% and 10% of its phase change. The double relaxation can be quantitatively analyzed by fitting the measured phase change with following equation [35]:

(8) $$\delta \left(t\right)=A\times e^{-\frac{t}{\tau_{1}}}+B\times e^{-\frac{t}{\tau_{2}}}$$

In Equation (8), the first term indicates the fast relaxation process, and the second term represents the slower one. Here, (*A*, *B*) and $(\tau_{1}$, $\tau_{2}$) are the corresponding weights and time constants. The ratio *A*/(*A* + *B*) stands for the degree of double relaxation. For example, when *A*/(*A* + *B*) = 1 (i.e., *B* = 0), it means double relaxation does not exist because of the vanishing second term. On the other hand, a larger B leads to a smaller *A*/(*A* + *B*), indicating a stronger double relaxation. 

In Figure 5, we use the single and double exponential decays to fit the experimental data. The fitting parameters *A*, *B*, $\tau_{1}$, $\tau_{2}$, and the calculated A/(A+B) are listed in Table 4. The measured relaxation time of PNLC-4 and HTG-4 is 0.57ms and 0.61ms at 25 °C, respectively.

### 3.3. Hysteresis

For some applications, such as beam steering, the employed PNLC device is expected to change the phase between 0 and 2π continuously. Hysteresis plays a critical role in grayscale control accuracy. In general, when a high voltage is applied to PNLC, the LC directors will be reoriented by the electric field. If the electric field is too high, then the polymer network could be deformed irreversibly, which is analogous to the stretching of a rubber band. Such a strong interaction between LC host and polymer network causes hysteresis. From experiment, we find that hysteresis increases with the electric field strength. Moreover, increasing the temperature can suppress the hysteresis effectively. 

**Figure 6:**
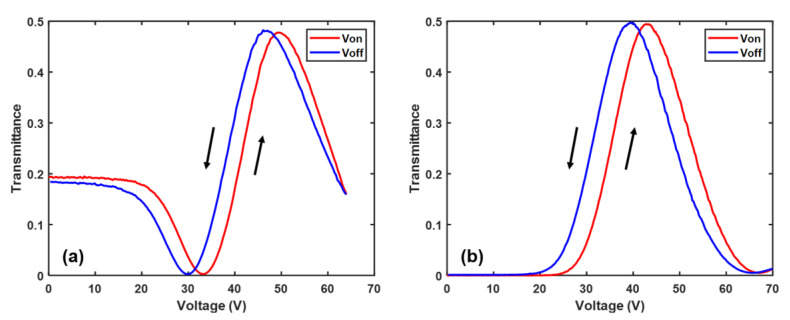
Forward and backward scans of voltage-dependent transmittance (VT) curves of (**a**) PNLC-4 and (**b**) HTG-4. The operating temperature is 25 °C.

Figure 6a,b shows the forward and backward scans of VT curves of PNLC-4 and HTG-4, respectively. The hysteresis is calculated from

(9) $$\Delta H=2\frac{\left(V_{\pi f}-V_{\pi b}\right)}{\left(V_{\pi f}+V_{\pi b}\right)}$$

In Equation (9), $V_{\pi f}$ is the forward voltage achieving π phase change and $V_{\pi b}$ is the backward voltage achieving π phase change. The hysteresis of PNLC-4 and HTG-4 at 25 °C is 7.0% and 8.7%, respectively. The reason PNLC-4 exhibits a slightly smaller hysteresis is due to the lower viscosity of M1, which in turn leads to a weaker interface interaction between the LC directors and the neighboring polymer networks. From Figure 6a, the initial and final transmittance at V=0 do not coincide perfectly. It takes few seconds to recover. This phenomenon is called residual birefringence. 

**Figure 7:**
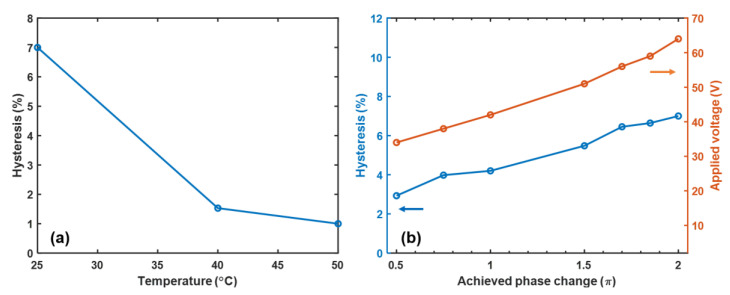
(**a**) Temperature dependent hysteresis of PNLC-4. (**b**) Hysteresis vs. applied voltage of PNLC-4 at 25 °C under different phase levels.

To investigate the hysteresis behavior at elevated temperatures, we chose PNLC-4 to conduct the experiment. An elevated temperature helps suppress hysteresis because the interaction between the LC molecules and polymer network is weaker due to the lower viscosity. Figure 7a shows the dramatically decreased hysteresis as the temperature increases. The hysteresis is suppressed from 7% at 25 °C to 1.5% at 40 °C and 1% at 50 °C. Additionally, we measured the hysteresis at 25 °C under different phase levels by controlling the applied voltages. Results are plotted in Figure 7b. At a low phase level, the hysteresis is less obvious due to the weaker interaction between LC and polymer network by smaller applied voltage.

### 3.4. Frequency Effect

**Figure 8:**
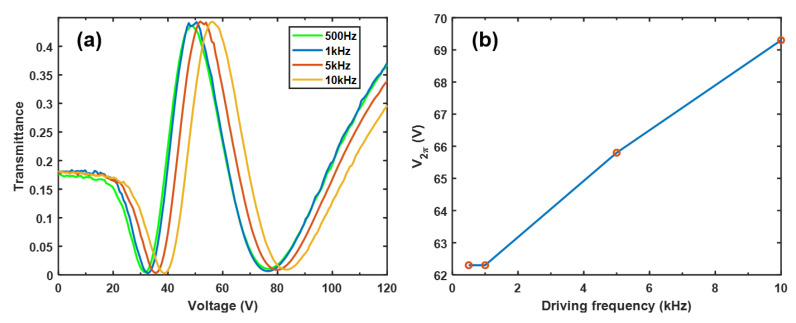
Measured (**a**) VT curves and (**b**) $V_{2\pi }$ of PNLC-4 at different driving frequencies.

For a high Δε LC, its Δ*ε* decreases as the electric field frequency increases. This phenomenon is known as dielectric relaxation [36], and a common example is dual-frequency LC materials [37]. To investigate the dielectric relaxation of PNLC-4, we measured its VT curves at different driving frequency (square waves) as shown in Figure 8. The VT curves overlap well when the driving frequency changes from 500 Hz to 1 kHz. As the frequency increases to 5 kHz and 10 kHz, the VT curve shifts toward right side, showing a higher $V_{th}$ and higher $V_{2\pi }$. The dielectric anisotropy is defined as $\Delta \varepsilon =\varepsilon_{//}-\varepsilon_{⊥}.$ The vertical dielectric constant ($\varepsilon_{⊥}$) is insensitive to the frequency, but the parallel component ($\varepsilon_{//}$) is highly dependent on the driving frequency, especially for the large Δε LCs. This is because a large Δε LC usually exhibits a high viscosity. When it is driven by a high frequency electric field, the LC directors cannot follow, resulting in a smaller effective Δε. 

### 3.5. Transmittance

**Figure 9:**
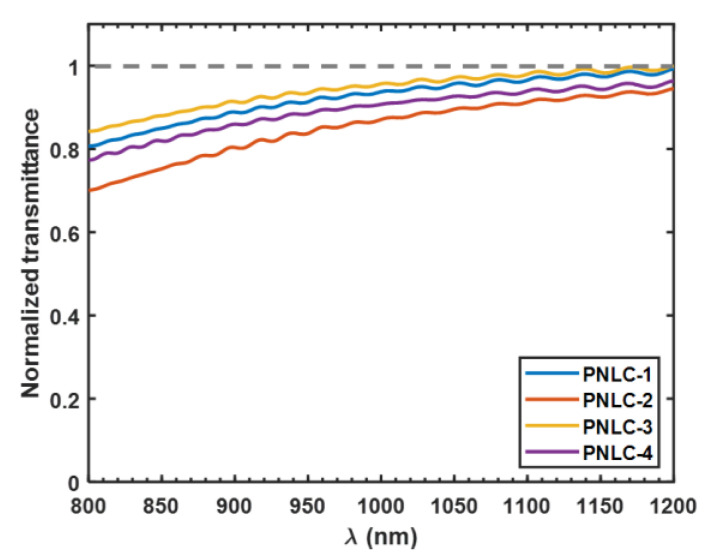
Normalized transmission spectra of PNLC 1–4 for an unpolarized light at 70 V.

In practical applications, high transmittance is critical. To analyze the scattering loss, we scan the transmittance of PNLC 1–4 from 800 to 1200 nm. The scattering loss of PNLCs can be analyzed by the Rayleigh–Gans–Debye model [22]. In this model, the transmittance of PNLC for a randomly polarized light at $V_{max}$ (where light scattering reaches maximum) is written as

(10) $$T=\frac{1}{2}\exp\left(-\frac{C_{e}\Delta n^{2}}{\lambda_{0}^{2}}d\right)+\frac{1}{2}\exp\left(-\frac{C_{o}\Delta n^{2}}{\lambda_{0}^{2}}d\right),$$

where $C_{e}$ and $C_{o}$ represents the domain size parameter for e-ray and o-ray, respectively. From Equation (10), for a given LC material, the transmittance is dependent on domain size and cell gap across the wavelength. Therefore, to explore the domain size and cell gap effects on scattering loss, we measured the transmittance of chose PNLC 1–4 at V=70 V (nearby $V_{2\pi }$) and the results are plotted in Figure 9. A sample filled with nematic M1 was used as reference to normalize the transmittance. By comparing these curves of different samples in Figure 9, we find that PNLC-3 has the highest transmittance because of its smaller domain size (6.6% monomer) and thinner cell gap (~10 μm). On the other hand, PNLC-2 has the maximum light scattering loss because of its larger domain size (5.7% monomer) and thicker cell gap (~12 μm). When comparing PNLC-1 with PNLC-4, although PNLC-4 has a higher monomer concentration (i.e. smaller domain size), its cell gap is thicker, as a result, PNLC-1 has a slightly (~4%) higher transmittance than PNLC-4 at *λ* = 1.06 μm. In all, the measured transmittance curves of 4 samples with different monomer concentration and cell gap are consistent with the theoretical analysis based on Equation (10). A more detailed theoretical analysis based on different models has been discussed in [22]. The oscillation of measured transmittance curves is due to the Fabry–P$\overset{´}{e}$ rot interference from mismatched refractive index between indium tin oxide (ITO) electrodes and liquid crystal. The specific data of normalized transmittance at 1.06 μm are listed in Table 3. 

## 4. Discussion

So far, we have investigated the $V_{2\pi }$, relaxation time, hysteresis, frequency effect, and light scattering loss of the transmissive PNLC devices. In this section, we compare the PNLCs with different monomer concentrations and cell gaps for potential applications at *λ* = 1.06 μm. Table 3 lists the experimental data of PNLC 1–4 where the LC host is our new material M1. 

From Table 3, both monomer concentration and cell gap play important roles affecting the performance of a PNLC device. First, for a given LC host, its $V_{2\pi }$ is related to the domain size and cell gap. Hysteresis originates from the interaction between LC and polymer network, which is affected by domain size. By doping more monomer to form smaller domain size, the LC molecules are more tightly constrained within the domains, leading to a smaller hysteresis. This hypothesis is confirmed by comparing the experimental results of PNLC-1,2 and PNLC-3,4. As discussed above, a smaller domain size also contributes to a faster response time by suppressing the double relaxation. The data in Table 3 show that PNLC-1 and PNLC-2 with less monomer exhibit a slower relaxation time compared to those of PNLC-3 and PNLC-4. This can be explained by double relaxation caused by larger domain size. In practical applications, light scattering loss is undesirable and should be minimized. In all, the PNLC with more monomers and thinner cell gap has lower light scattering loss, as indicated by PNLC-3. From Table 3, there is a significant trade-off between monomer concentration and cell gap. For example, if high transmittance is top priority, then PNLC-3 is the best choice because of its lowest scattering loss, however, its $V_{2\pi }$ is compromised. On the other hand, if low $V_{2\pi }$ is preferred, then we may slightly sacrifice the light scattering loss or relaxation time depending on the application requirements. 

Next, we compare the electro-optical performance of PNLC-4 with HTG-4 because they have the same monomer concentration and cell gap; the only difference is different LC hosts. As Table 3 shows, PNLC-4 has a lower $V_{2\pi }$ than HTG-4 due to its larger Δn and Δε. It is noteworthy that both samples still suffer from ~7% to 8% scattering loss at 1.06 μm. One promising approach to suppress light scattering is to lower the curing temperature during polymerization process [22]. As demonstrated in [22], low temperature, e.g., 11 °C, increases the precursor’s viscosity dramatically, which slows down the monomer diffusion rate and leads to smaller domain sizes during polymerization process. As a result, light scattering is suppressed significantly. In all, our M1 based PNLC shows a lower operating voltage without compromising other performances when compared to those using HTG host.

Finally, it is worth mentioning that our above PNLCs are designed for transmissive mode operation. If we use a reflective LCoS, then we can obtain 2*π* phase change by using a 50% thinner cell gap because the incident light traverses the PNLC layer twice. As a result, its $V_{2\pi }$ should be reduced by ~2x, as Equation (5) and Figure 4 indicate.

## 5. Conclusions

In conclusion, we have developed a new LC mixture, designated as M1, which is a promising host for PNLCs to lower the operating voltage because of its high Δn and large Δε. The measured relaxation time is in the submillisecond regime. Moreover, we fabricated 4 PNLCs based on M1 mixture with different monomer concentrations and cell gaps to explore their electro-optical performances. A thicker cell gap and a lower monomer concentration help to reduce $V_{2\pi }$ but the tradeoffs are higher scattering loss and slower response time. On the contrary, a thinner cell gap and a higher monomer concentration help to reduce the response time and light scattering but the $V_{2\pi }$ is compromised. Compared with the commercial material HTG, M1’s birefringence is 10% higher without compromising dielectric anisotropy and viscosity. Our high Δ*n* M1 mixture is not limited to PNLCs but it is also applicable to polymer-stabilized blue phase liquid crystals. Potential applications of PNLCs for laser beam steering and spatial light modulators especially in the IR region are foreseeable. 

## Figures and Tables

**Table 1:** Measured physical properties of M1 and HTG-135200 (HTG) at *T* = 25 °C.

LC Mixture	M1	HTG-135200
*T_c_* (°C)	107.6	96.4
Δ*n* @1.06 μm	0.207	0.185
∆*ε* @1 kHz	85.9	73.4
$\gamma_{1}\left(\mathrm{mPa}\cdot S\right)$	1062	1080
*K*_11_ (pN)	12.1	11.3
*γ*_1_/*K*_11_ (ms/µm^2^)	87.9	96.0

**Table 2:** Fitting parameters obtained through Equations (2)–(4).

LC Host	$\Delta n_{0}$	*β*	*A* (ms/µm^2^)	*E_a_* (meV)	G $\left(\mu m^{-2}\right)$	$\lambda^{*}$ (μm)	$G\lambda^{*}{}^{2}$
M1	0.258	0.172	5.85 × 10^−5^	358.7	3.49	0.234	0.191
HTG	0.236	0.153	1.17 × 10^−5^	402.5	3.19	0.235	0.176

**Table 3:** Compositions and electro-optic performance of eight polymer-network liquid crystal (PNLC) samples with different RM257 concentrations and cell gaps. *λ* = 1.06 μm.

Sample	LC Host	RM257	Irg819	Cell Gap (μm)	$V_{2\pi }\;\left(V\right)$	Hysteresis	Relaxation Time (ms)	Transmittance
PNLC-1	M1	5.7%	0.5%	10.02	62.0	7.6%	~1.0	96.4%
PNLC-2	M1	5.7%	0.5%	12.15	58.2	7.9%	~1.0	89.8%
PNLC-3	M1	6.6%	0.5%	9.92	77.4	6.9%	0.80	98.3%
PNLC-4	M1	6.6%	0.5%	12.15	64.8	7.0%	0.61	92.5%
HTG-1	HTG	5.7%	0.5%	9.93	70.0	-	-	-
HTG-2	HTG	5.7%	0.5%	12.15	63.6	-	-	-
HTG-3	HTG	6.6%	0.5%	10.2	78.0	-	-	-
HTG-4	HTG	6.6%	0.5%	12.15	71.4	8.7%	0.57	93.2%

**Table 4:** Fitting parameters obtained through Equation (8).

Sample	*A*	*B*	$\tau_{1}\left(ms\right)$	$\tau_{2}\left(ms\right)$	A/(A+B)
PNLC-4	4.34	0.0728	0.22	15.05	98.4%
HTG-4	8.31	0.087	0.17	29.51	99.0%
